# Factors Associated with Household Transmission of Pandemic (H1N1) 2009 among Self-Quarantined Patients in Beijing, China

**DOI:** 10.1371/journal.pone.0077873

**Published:** 2013-10-18

**Authors:** Daitao Zhang, Wenting Liu, Peng Yang, Yi Zhang, Xinyu Li, Kaylyn E. Germ, Song Tang, Wenjie Sun, Quanyi Wang

**Affiliations:** 1 Beijing Center for Disease Prevention and Control, Beijing, China; 2 The Institute of Environmental and Human Health, Texas Tech University, Lubbock, Texas, United States of America; 3 Department of Global Environmental Health Sciences, School of Public Health and Tropical Medicine, Tulane University, New Orleans, Louisiana, United States of America; University of Florida, United States of America

## Abstract

As the pandemic (H1N1) 2009 progressed, the Ministry of Health of China advised cases with mild symptoms to remain home for isolation and observation, which may have increased the risk for infection among other household members. Describing the transmission characteristics of this novel virus is indispensable to effectively controlling the spread of disease; thus, the aim of this study was to assess risk factors associated with household transmission of pandemic H1N1 from self-quarantined patients in Beijing, the capital city of China. A 1:2 case-control study with 54 case households and 108 control households was conducted between August 1 and September 30, 2009 in Beijing. Cases were households with a self-quarantined index patient and a secondary case, while controls were households with a self-quarantined index patient and a close contact. Controls were also matched to cases for sex and age of index case-patient. A structured interview guide was used to collect the data. Conditional logistical models were employed to estimate Odds Ratios (OR) with 95% confidence intervals (95% CI). Results indicated that higher education level (OR 0.42; 95% CI 0.22-0.83), sharing room with an index case-patient (OR 3.29; 95%CI 1.23-8.78), daily room ventilation (OR 0.28; 95%CI 0.08-0.93), and hand washing ≥3/d (OR 0.71; 95%CI 0.48-0.94) were related to the household transmission of pandemic H1N1 from self-quarantined patients. These results highlight that health education, as well as the quarantine of the index case-patient immediately after infection, frequent hand hygiene, and ventilation are critical to mitigating household spread of pandemic H1N1 virus and minimizing its impact. Household contacts should be educated to promote these in-home practices to contain transmission, particularly when household members are quarantined at home.

## Introduction

On May 16, 2009, the first case of pandemic (H1N1) 2009 was diagnosed in Beijing, China. The majority of people with pandemic (H1N1) 2009 experienced mild illness or recessive infection[1]. Subjecting others to possible infection and unknown transmission of the virus may occur in a confined setting, such as households, hospitals, universities, communities, or other public places[2,3]. It has been reported that the secondary attack rate (SAR) of pandemic (H1N1) 2009 was 10% to 45%[4-7], and by the end of 2009, the total number of confirmed cases in Beijing had sharply risen to 10,802.

Isolation or quarantine of index case-patients is an effective strategy to prevent and contain the incidence and spread of influenza pandemic[8,9]. From May to June 2009, the confirmed case-patients in Beijing were quarantined in designated hospitals to receive treatment; however, on July 14, 2009, the Ministry of Health of China advised those who had mild symptoms to stay at home for isolation and observation, which put other household members at a higher risk for infection[10,11]. Household transmission of pandemic (H1N1) 2009 has the potential to trigger acommunity transmission and even a nationwide pandemic[7]. Previous studies have demonstrated that household transmission has accounted for up to 40% of all cases detected[12], and the occurrence of confirmed infection was >5% via close household contacts in Beijing[13].

 Reducing the number of secondary cases in the household is essential to minimizing the impact of influenza. Recent studies have begun to identify key epidemiological characteristics of household transmission of pandemic (H1N1) 2009[6,14-18]. A variety of household-level risk factors have been identified including age of index patient, size of the home, number of household members, exposure during the index patient’s symptomatic phase, as well as medication initiated from the onset of fever[7,13,16,19]. These studies mainly used a prospective cohort approach. Entry into the cohort required a confirmed infection of one household member and was analyzed on an individual basis. The secondary infection cases often lacked the support of laboratory confirmation, and therefore led to potential misestimates of the SAR. Additionally, a very recent systematic review revealed that the variation of household transmissibility of pandemic (H1N1) 2009 was partly due to the differences in study design[20].

 In the present study, we formulated a 1:2 retrospective case-control study to unravel possible risk factors associated with household transmission of pandemic (H1N1) 2009 from index patients who were voluntarily quarantined at home during pandemics in 2009 in Beijing. This study allowed further investigation into previous household findings of influenza transmission and provided effective strategies for future prevention and control measures of respiratory epidemic risk.

## Methods

From July to October of 2009, as the pandemic progressed, the Ministry of Health of China advised those who had mild symptoms to stay at home for isolation and observation. During this period, H1N1 influenza infections did not reach an epidemic peak in Beijing, and any person suspected of infection could be interviewed by a Beijing or local CDC staff member. The information was then reported to the Beijing CDC via the online reporting system (China Information System for Diseases Control and Prevention). At the same time, a swab of both tonsils and the posterior pharyngeal wall were collected. The throat swab was sent to 38 network laboratories and tested by real-time reverse transcriptase polymerase chain reaction (RT-PCR) for pandemic influenza A (H1N1). The test results were recorded in the same online system as required by law.

A detailed epidemiological investigation was then conducted for each laboratory-confirmed case of pandemic (H1N1) 2009 infection (including symptomatic and asymptomatic cases) by Beijing and local CDCs within 6 hours of infection confirmation. Index case-patients and their household members were interviewed about the occurrence of illness, their medical history, and their health habits. Respiratory samples were also collected from all members of identified households early in the investigation regardless of respiratory symptoms. All household contacts were traced and quarantined for 7 days after the last exposure to the index case-patient. A second pharyngeal swab specimen was collected to test for pandemic (H1N1) 2009 virus to investigate if any of the symptoms (axillary temperature ≥ 37.3°C, cough, sore throat, nasal congestion, and rhinorrhea) developed from household contacts during quarantine.

### Study Design

 According to the inclusion standards, the data of households with index case-patient detected between August 1 and September 30, 2009 across 6 districts in Beijing were collected from an online reporting system. All patient cases were confirmed by RT-PCR for pandemic influenza A (H1N1). Households were then divided into two groups according to the following inclusion standard of case households and control households. The case households and control households were 1:2 matched in order to increase statistical power. The index patients in control households were matched to the index patients in case households by sex and age (±5 years). In total, 54 case households (an index case-patient and a secondary case per household) and 108 control households (an index case-patient and a closest contact per household) were enrolled. In the present study, the closest contact had the highest contact-frequency and contact-time with index case-patient among all family members per control household.

### Study participants

 Case households were recruited by following criteria: (1) has an index patient of pandemic (H1N1) 2009 influenza in the household between August 1 to September 30, 2009; (2) index case was quarantined in the household from the onset of diagnosis to 7 days after the onset of illness; (3) the secondary case had potential contact with the index patient; (4) the symptoms onset of secondary case occurred within 7 days since the last known contact with the index case during the infectious period of the index case; (5) the RT-PCR confirmation date of secondary case occurred within 7 days since last known contact with the index case during the infectious period of index case; and (6) none of the household members previously received a vaccine against pandemic (H1N1) 2009 influenza.

 Case households were excluded if: (1) the index case-patients were quarantined in hospital, or stayed in the household alone, whereby they were not in contact with family members; and (2) the secondary cases were transmitted by others from outside of the household.

Control households were recruited by: (1) had an index patient of pandemic (H1N1) 2009 influenza in the household between August 1 to September 30, 2009; (2) index cases were quarantined in the household from the date of diagnosis to 7 days after the onset of illness; (3) their family members had potential close-contact with the index patient; (4) none of the closest contact had been laboratory-diagnosed as having pandemic (H1N1) 2009 influenza during the transmission period of index patient; and (5) none of the household members previously received a vaccine against pandemic (H1N1) 2009 influenza.

### Data collection

The investigators found the addresses of eligible case and control households through the “China Information System for Disease Control and Prevention”, and subsequently carried out household surveys. Epidemiologists and trained CDC staff conducted standardized and structured interviews to gather related information through validated questionnaires. “The questionnaire for the index case-patients of pandemic (H1N1) 2009” and “the questionnaire for the secondary cases and close contacts of pandemic (H1N1) 2009” were originally designed to collect basic pandemic (H1N1) 2009 research including household-level characteristics (household income, size, living space, etc.), information of index case-patient, secondary case, and close contacts (age, sex, occupation, education level, relationship, etc.), and epidemic risk factors (contact time and frequency, signs and symptoms, date of symptom onset, health habits, influenza vaccination, etc.).

### Statistical analysis

Statistical analysis was performed using SPSS11.5 statistical package (SPSS Inc., Chicago, Illinois, USA). Median and range values were calculated for continuous variables, and percentages were calculated for categorical variables. Differences in characteristics of household-level, index case-patient, and secondary case (closest contact) between case and control households were assessed via either a chi-square test or Fisher’s exact test. The risk factors identified above (at *p*<0.10 level) were then evaluated for independent associations via conditional logistic regression. All *p* values were based on a two-sided test of statistical significance. Significance was accepted at the level of *p*≤0.05 in multivariate analysis.

### Ethic Statement

 This study was approved by the Institutional Review Board and Human Research Ethics Committee of Beijing Center for Disease Prevention and Control. Written informed consent was obtained from each adult participant or guardian of the child participant.

## Results

### Characteristics of case households and control households

There were no statistical differences in household-level characteristics such as total household income (*p*=0.285) and living space of family per capita (*p*=0.413), between case and control households (Table 1). However, a significant difference was observed in household size (*p*=0.055) between case and control households at *p*<0.1 level.

**Table 1:** Characteristics of household, index case, and secondary case (closest contact) in this study.

**Characteristics**	**Category**	**Subcategory**	**Case household**	**Control household**	***P* value**
			**No. (%)**	**No. (%)**	
**Household-level**	Total household income (RMB/months)	<2000	11(20.3)	9(8.3)	0.285
		2000-4999	14(25.9)	39(36.1)	
		5000-9999	21(38.9)	44(40.7)	
		≥10000	8(14.8)	16(14.8)	
	Household size	≤3 member	26(48.1)	69(63.9)	**0.055**
		≥4 member	28(51.9)	39(36.1)	
	Living space of family per capita	<20m^2^	3(5.6)	10(9.3)	0.413
		≥20m^2^	51(94.4)	98(90.7)	
**Index case**	Age(years)	0-9	9(16.7)	20(18.5)	0.967*
		10-19	25(46.3)	48(44.4)	
		20-29	11(20.4)	23(21.3)	
		30-39	6(11.1)	11(10.2)	
		40-49	1(1.9)	4(3.7)	
		≥50	2(3.7)	2(1.9)	
	Time of hospital visiting	≤48h after illness onset	47(87.0)	96(88.9)	0.666
		>48h after illness onset	4(7.4)	9(8.3)	
	Signs and symptoms	No visit to the hospital	3(5.6)	3(2.8)	
		Fever	45(83.3)	95(88.1)	0.417
		Cough	37(68.5)	56(51.9)	**0.043**
		Sore throat	24(44.4)	43(39.8)	0.573
		Runny nose	13(24.1)	24(22.2)	0.791
		Sneeze	7(13.0)	14(13.0)	1
		Headache	10(18.5)	15(13.9)	0.442
		Myalgia	6(11.1)	13(12.1)	0.863
	Covering with hands when coughing or sneezing		38(70.4)	78(72.2)	0.805
	Avoid contacting family members when illness onset		27(50.0)	66(61.1)	0.178
	Frequency of hand washing (≥3/d)		46(85.2)	94(87.0)	0.746
	Mask wearing every day		28(51.2)	29(24.1)	0.656
**Secondary case or**	Age(years)	0-9	5(9.3)	2(1.9)	**0.086** *
**closest contact**		10-19	6(11.1)	5(4.6)	
		20-29	7(13.0)	8(7.4)	
		30-39	14(25.9)	39(36.1)	
		40-49	17(31.5)	41(38.0)	
		≥50	5(9.2)	13(12.0)	
	Education level	Middle school and lower	15(27.8)	12(11.1)	**0.007**
		High school and higher	39(72.2)	96(88.9)	
	Frequency of hand washing (≥3/d)		41(75.9)	105(97.2)	**<0.001**
	Mask wearing every day		23(42.6)	47(43.5)	0.911
	Sterilizing the room (UV or disinfectants)		6(11.1)	15(13.9)	0.62
	Sharing room with index case-patient		38(70.4)	50(46.3)	**0.004**
	Ventilating room every day		38(70.4)	99(91.7)	**<0.001**
	Relationship to index case-patient	Parents or children	36(66.7)	60(55.6)	0.292
		Couples	10(18.5)	32(29.6)	
		Friends	8(14.8)	16(14.8)	

* Fisher exact test

For index cases, there were no significant differences between case and control groups in: the time of hospital visiting after illness onset (*p*=0.666), covering with hands when coughing or sneezing (*p*=0.805), avoiding contact with family members after the onset of illness (*p*=0.178), frequent hand washing (≥3/d) (*p*=0.746), and mask wearing (*p*=0.656) (Table 1). Also, no statistically significant differences were found with other symptoms such as fever, sore throat, runny nose, sneeze, headache, and myalgia (*p*>0.1). However, the cough rate of index cases in case households was 68.5%, which was significantly higher than that of index cases in control households (51.9%) (*p*=0.043).

 For the secondary cases in case households and the closest contacts in control households, there were no significant differences in mask wearing (*p*=0.911), room sterilization (*p*=0.62), and relationship to index case-patient (*p*=0.292) (Table 1). However, the proportion of hand washing occurring more than 3 times per day of closest contact in control groups (97.2%) was significantly higher than that of secondary cases in case group (75.9%) (*p*<0.001). In addition, compared to the closest contacts from control households, the percentage of sharing room with index case-patient of secondary cases in case household was significantly higher (*p*=0.004), while the proportion of ventilation of the areas every day was significantly lower (*p*<0.001).

### Factors Associated with Household Transmission of Pandemic (H1N1) 2009

The risk factors that were statistically different between case and control groups (*p*<0.1), as shown in Table 1, and which were related to the transmission of pandemic (H1N1) 2009, were enrolled in multivariate analysis. As a result, seven factors were selected, specifically: (1) household size, (2) cough of index case, (3) age of secondary cases and closest contacts, (4) education level of secondary cases (closest contact), (5) whether hand-washing occurred regularly, (6) sharing room with an index case, and (7) ventilation of the room(s). By using multivariate logistic regression analysis, four statistically significant independent risk factors associated with household transmission of pandemic (H1N1) 2009 were identified (*p*≤0.05), including education level of high school and higher (OR=0.42; *p*=0.01), sharing room with an index case (OR=3.29; *p*=0.02), ventilating the room every day (OR=0.28; *p*=0.04), and hand washing frequency ≥3 times/d (OR=0.71; *p*=0.05) (Table 2).

**Table 2:** Factors significantly associated with household transmission of pandemic (H1N1) 2009 using multivariate analysis.

**Factors**	**OR (95%CI)**	***P* value**
Education level of secondary case (closest contact)		
Middle school and lower	Reference	
High school and higher	0.42 (0.22-0.83)	0.01
Sharing room with index case-patient	3.29 (1.23-8.78)	0.02
Ventilating room every day	0.28 (0.08-0.93)	0.04
Frequency of hand washing		
<3/d	Reference	
≥3/d	0.71 (0.48-0.94)	0.05

## Discussion

This study reports on households with index case-patients with H1N1 influenza detected between August 1 and September 30, 2009 across 6 districts in Beijing, China. The results reflect the transmission of pandemic (H1N1) 2009 in the absence of vaccine intervention and provide a pure estimate which more closely characterizes transmission without the influence of vaccine. To our knowledge, this is the first matched case-control study to evaluate the household transmission dynamics of pandemic (H1N1) 2009. In the present study, univariate analysis was used to evaluate the effect of all factors individually, while multivariable logistical regression was applied to eliminate confounding factors and determine significant risk factors in household transmission.

Our results indicate that the risk of pandemic (H1N1) 2009 transmission was reduced as the household education-level increased, which is likely due to people with a high education level being more likely to adopt appropriate viral counter-measures to protect themselves from infection. We found that household size and sharing a room with index case-patient were both risk factors determined by univariate analysis. These data reveal that those particular housing conditions may be associated with spreading pandemic (H1N1) 2009 within households. In an investigation conducted by Loustalot et al.[19], authors found that secondary infection rates of pandemic (H1N1) 2009 within households were increased with the number of persons residing in the household and rates decreased with an increasing number of rooms. These findings are in accordance with our results indicating that housing density plays an essential role in the household transmission of the pandemic (H1N1) 2009; moreover, multivariable logistic regression analysis showed that sharing a room with an index case was an independent risk factor of being infected by pandemic (H1N1) 2009. Compared to close contacts living in a single room, the risk of infection of those sharing a room with the index case-patient was 3.29 times greater. As the factor of household size is related to sharing the room with index case, it was not included in multivariate analysis.

Ventilating the room and household every day, as well as hand washing ≥3 times/d were found to be protective factors in this study. As pandemic (H1N1) 2009 is a respiratory disease and is easily transferred through close contact, contaminated hands can serve as vehicles of transmission of upper-respiratory illness (URI)[21]. Proper hand hygiene, as well as wearing gloves and masks can effectively control the spread of respiratory disease within a household[22-25]. On these grounds, strengthening public health education and promotion of proper personal hygiene practices may have a positive impact on targeted control measures of pandemic (H1N1) 2009 transmission prevention.

Although predicted risk factors such as covering coughs, non-parent relationship of index-cases, timely treatment of index case-patients, as well as using antiviral prophylaxis were included in our study, we did not observe any decreased individual risk of pandemic (H1N1) 2009. This is contrary to previous studies suggesting that these are protective factors of pandemic (H1N1) 2009 transmission[5]. This may be explained by small sample size and the chosen design of the current study.

Our study does contain certain limitations. The first limitation is recall bias. As designed, this study was conducted in May of 2010, and the data for both cases and controls, was collected retrospectively, most notably the exposure information. Other than non-differential and imperfect recall, controls maybe more likely to have forgotten about their high-risk behaviours than in infection cases. Secondly, a household misclassification bias may have occurred within the analysis because some influenza infections acquired in the household could have been asymptomatic[26] and not all infections would be laboratory confirmed. Even with intensive surveillance, some of the control households may have been incorrectly classified. Thirdly, the results were inferred based on the self-quarantined population, limiting the generalization. The degree to which individuals complied with the quarantine recommendations may have influenced our conclusions.

In conclusion, the results of this study give evidence that it is essential to strengthen health education, promote attention toward personal hygiene, and advocate appropriate household ventilation in order to efficiently protect against influenza transmission.
